# Senile Systemic Amyloidosis: Clinical Features at Presentation and Outcome

**DOI:** 10.1161/JAHA.113.000098

**Published:** 2013-04-24

**Authors:** Jennifer H. Pinney, Carol J. Whelan, Aviva Petrie, Jason Dungu, Sanjay M. Banypersad, Prayman Sattianayagam, Ashutosh Wechalekar, Simon D. J. Gibbs, Christopher P. Venner, Nancy Wassef, Carolyn A. McCarthy, Janet A. Gilbertson, Dorota Rowczenio, Philip N. Hawkins, Julian D. Gillmore, Helen J. Lachmann

**Affiliations:** 1 National Amyloidosis Centre, UCL Medical School, Royal Free Hospital, London, UK (J.H.P., C.J.W., J.D., S.M.B., P.S., A.W., S.J.G., C.P.V., N.W., C.A.M.C., J.A.G., D.R., P.N.H., J.D.G., H.J.L.); 2 UCL Centre for Nephrology, UCL Medical School, Royal Free Hospital, London, UK (J.H.P., J.D.G., H.J.L.); 3 Biostatistics Unit, UCL Eastman Dental Institute, London, UK (A.P.); 4 St George's Hospital University of London, London, UK (J.D.); 5 The Heart Hospital UCLH United Kingdom, London, UK (S.M.B.)

**Keywords:** amyloid, heart failure, prognostic factors, survival, transthyretin

## Abstract

**Background:**

Cardiac amyloidosis is a fatal disease whose prognosis and treatment rely on identification of the amyloid type. In our aging population transthyretin amyloidosis (ATTRwt) is common and must be differentiated from other amyloid types. We report the clinical presentation, natural history, and prognostic features of ATTRwt compared with cardiac‐isolated AL amyloidosis and calculate the probability of disease diagnosis of ATTRwt from baseline factors.

**Methods and Results:**

All patients with biopsy‐proven ATTRwt (102 cases) and isolated cardiac AL (36 cases) seen from 2002 to 2011 at the UK National Amyloidosis Center were included. Median survival from the onset of symptoms was 6.07 years in the ATTRwt group and 1.7 years in the AL group. Positive troponin, a pacemaker, and increasing New York Heart Association (NYHA) class were associated with worse survival in ATTRwt patients on univariate analysis. All patients with isolated cardiac AL and 24.1% of patients with ATTRwt had evidence of a plasma cell dyscrasia. Older age and lower N‐terminal pro‐B‐type natriuretic peptide (NT pro‐BNP) were factors significantly associated with ATTRwt. Patients aged 70 years and younger with an NT pro‐BNP <183 pmol/L were more likely to have ATTRwt, as were patients older than 70 years with an NT pro‐BNP <1420 pmol/L.

**Conclusions:**

Factors at baseline associated with a worse outcome in ATTRwt are positive troponin T, a pacemaker, and NYHA class IV symptoms. The age of the patient at diagnosis and NT pro‐BNP level can aid in distinguishing ATTRwt from AL amyloidosis.

## Introduction

Without effective treatment cardiac amyloidosis is a fatal disease. There are 3 major amyloid types that affect the heart: light‐chain (AL) amyloidosis, hereditary transthyretin amyloidosis (ATTRm), and wild‐type transthyretin amyloidosis (ATTRwt), also known as senile cardiac amyloidosis.^1–3^ Correctly identifying the amyloid type is vital, as it has a major impact on prognosis and completely dictates treatment. Biopsy followed by Congo Red staining and immunohistochemistry is the gold standard for diagnosing and typing amyloid.^4^ Correct diagnosis of isolated cardiac amyloidosis clinically is especially challenging as symptoms are often nonspecific. The diagnosis of ATTRm is relatively straightforward if genetic screening of the *TTR* gene is performed, but differentiating between ATTRwt and AL amyloidosis can be more problematic. ATTRwt invariably affects older patients, who may well have an incidental monoclonal gammopathy of undetermined significance (MGUS). If the coexistence of amyloidosis and a plasma cell dyscrasia alone is used as evidence of AL amyloidosis, chemotherapy‐based treatment may be given in error.^5^

Recent advances in cardiac magnetic resonance imaging (MRI) have led to a dramatic increase in detection of late gadolinium enhancement, which is reported to be characteristic of amyloidosis. Some features appear more characteristic of ATTR amyloid such as transmural late gadolinium enhancement (LGE) and right ventricular LGE, but currently MRI appearances cannot differentiate between amyloid types.^6^ ATTRwt amyloidosis is almost certainly underdiagnosed,^7^ and as the prevalence increases with age and the population demographic shifts toward the elderly, the burden of disease will become an increasing problem.^8–9^

Currently there is no specific licensed treatment for ATTRwt amyloidosis, but new drugs are on the horizon, with several clinical trials currently recruiting patients with transthyretin amyloidosis (http://clinicaltrials.gov/ct2/results?term=transthyretin). Defining the clinical course of the disease is important both for patient counseling for a disease for which there is currently limited literature and to identify clinical diagnostic and response criteria for further treatment trials.^10–11^

Here we report the clinical presentation of 102 patients with ATTRwt seen at the UK amyloid referral center between 2002 and August 2011 and compare them to 36 patients with isolated cardiac AL amyloidosis. We describe the natural history of the disease and identify baseline factors that are associated with poor survival. By comparing patients with ATTRwt and isolated cardiac AL amyloidosis, we have created a model to calculate the probability of a diagnosis of ATTRwt amyloidosis. From this model we have designed a diagnostic algorithm that can help to guide a diagnostic approach.

## Methods

### Patients

We analyzed all 102 patients with biopsy‐confirmed ATTRwt diagnosed between 2002 and August 2011 at a single UK center and compared them with the 36 patients with biopsy‐confirmed isolated cardiac AL amyloidosis diagnosed over the same period at the same center.

Patients were referred from throughout the United Kingdom and Ireland. All referrals were accepted irrespective of the previous investigations or prior evidence of amyloid. In the AL group, 20 patients were referred from cardiology, and 16 patients were referred by hematologists; all AL patients had been seen by a cardiologist before initial assessment at our center. In the ATTRwt group, 73 patients were referred from cardiology, 18 from hematology, 3 from general practice, 2 each from gastroenterology and urology, and 1 each from elderly care, neurology, rheumatology, and respiratory medicine. Ninety‐three of the 102 patients in the ATTRwt group had been seen before referral by a cardiologist.

At our center all patients had an initial diagnostic assessment that included clinical evaluation, ECG, echocardiogram, serum amyloid P component (SAP) scintigraphy, and biochemical evaluation. Onset of symptoms was defined on the basis of the history, but a diagnosis of amyloidosis was dated from the biopsy, which provided histological proof of amyloid. If necessary after our initial assessment, advice was given to the referring doctor regarding further investigations required to confirm the diagnosis histologically. Patients were then reviewed at 6 to 12 monthly intervals. Follow‐up assessment comprised clinical and biochemical evaluation, ECG, and echocardiogram. N‐terminal pro‐B‐type natriuretic peptide (NT pro‐BNP) concentration was measured in all patients presenting after 2007. In patients diagnosed earlier, NT pro‐BNP was measured on banked serum samples that had been kept at −30°C. Evidence of a plasma cell dyscrasia was defined as any of the following: detectable M‐protein on plasma electrophoresis or immunofixation, Bence Jones proteins in urine, or abnormal serum free light‐chain level (kappa >19.4, lambda >26.3) or abnormal ratio (0.26 to 1.65).

The study was approved by the Ethics Committee of the Royal Free Hospital, and patients provided informed consent.

### Diagnostic Procedures

Amyloid deposition was confirmed histologically in all patients; sections from formalin‐fixed, paraffin‐embedded biopsy specimens were stained with Congo Red and viewed under cross‐polarized light.^4^ Immunohistochemical staining of amyloid deposits was performed using monospecific antibodies reactive with SAA, kappa, lambda, and transthyretin as previously described.^12^ A diagnosis of AL amyloidosis was only made if there was positive kappa or lambda immunohistochemical staining of amyloid in endomyocardial tissue. All the ATTRwt patients had immunohistochemical confirmation of transthyretin amyloidosis from a variety of tissues.

All patients with transthyretin amyloidosis were confirmed to be wild type on genotyping. Genomic DNA was extracted from whole blood treated with EDTA.^13^ The coding regions of the transthyretin gene were amplified by polymerase chain reaction assay. Exons 2, 3, and 4 of the *TTR* gene were sequenced as previously described.^14^

All patients had amyloidotic cardiomyopathy defined by either endomyocardial biopsy proof of amyloid deposition or a mean left ventricular wall thickness (septum and posterior wall) >12 mm in the absence of hypertension or other causes of left ventricular hypertrophy, consistent with published consensus criteria for AL amyloidosis.^15^ As there is currently no published consensus for ATTR amyloid, the same criteria were used.

Significant systemic amyloidosis was excluded by whole‐body anterior and posterior scintigraphic imaging after administration of ^123^I‐labeled SAP using an Elscint Superhelix gamma camera was undertaken at baseline.^16^

### Instrumental Definitions

ECG measures were based on standard definitions.^17^ Echocardiographic measures of chamber quantification were based on standard recommendations.^18^ In the survival analysis left ventricular wall thickness was graded as mild 1.3 to 1.5 cm, moderate 1.6 to 1.9 cm, and severe >2.0 cm. Ejection fraction was graded as normal >55%, mild impairment 45% to 54%, moderate impairment 36% to 44%, and severe impairment <35%.

Classification of diastolic dysfunction on echocardiography was based on published data.^19^ Not all patients were included in the classification of diastolic dysfunction because of lack of measured parameters in patients diagnosed before 2005.

### Statistical Analysis

Results are expressed as mean (±SD), median (Q1, Q3), or percentage as appropriate. Patient follow‐up was censored at the last clinic visit. Univariate analysis compared the baseline data in the 2 groups used the unpaired *t* test (if the data were normally distributed) and the nonparametric Mann–Whitney test for numerical variables and the chi‐square or Fisher exact test for categorical variables. Factors that achieved statistical significance (*P*<0.05) and were deemed clinically relevant were included in a multivariable logistic regression model (SPSS Statistics version 19).

Patient survival was estimated by Kaplan–Meier analysis (Stata version 11; StataCorp LP). The log‐rank test was used to compare differences in stratified Kaplan–Meier survival curves. Cox regression analysis using a backward stepwise approach was used to investigate factors associated with overall survival in ATTRwt patients, using IBM SPSS Statistics (version 19); statistical significance was achieved if *P*<0.05.

## Results

### Referral Patterns and Diagnosis

Of the 102 diagnosed with ATTRwt; 65 (63.7%) were diagnosed on endomyocardial biopsy, 24 (23.5%) on gastrointestinal biopsy, 4 (3.9%) from bladder biopsy, 4 (3.9%) on fat biopsy, 1 (0.9%) on carpal tunnel biopsy, and 1 (0.9%) from fallopian tube tissue. Fifteen patients (41.6%) in the AL group and 46 patients (46.5%) in the ATTRwt group had amyloid proven on a biopsy before the initial assessment. The initial investigation that prompted the referral or biopsy in the AL group was echocardiogram in 22 (61.1%), cardiac magnetic resonance (CMR) imaging in 12 (33.35%), and bone marrow and CT scan in 1 each. In the ATTRwt group the initial investigation that prompted referral or biopsy was echocardiogram in 55 (55.5%), CMR in 37 (37.4%), cystoscopy in 2 cases, and colonoscopy, gastroscopy, and cardiomegaly on chest x‐ray in 1 each. Two cases were referred on the basis of evidence of a plasma cell dyscrasia and a history of breathlessness. Thirty‐six of a total of 1953 patients with AL amyloidosis diagnosed with isolated cardiac AL amyloidosis after comprehensive evaluation represented 1.84% of AL patients.

The number of diagnoses of ATTRwt has increased in the past 5 years, with a doubling in the last 12 months, whereas the numbers of patients with isolated cardiac AL amyloidosis has remained the same (data not shown). In the ATTRwt group from 2007 to 2011, 75% to 80% of referrals each year were from cardiologists.

### Baseline Characteristics

#### Features in the history

Three patients did not have evidence of cardiac amyloid on echocardiography. These were patients with amyloid in tissue from the gastrointestinal tract, bladder, and carpal tunnel; these patients were therefore removed from further analyses.

Baseline (initial assessment) patient demographics of both groups can be seen in Table 1. ATTRwt is a predominantly male disease; 88.8% of affected patients were men, significantly higher than the 69.4% of cardiac AL patients. The median age at presentation was 73 years, significantly older than the 63 years in cardiac AL. Presenting symptoms were similar in both groups, with the majority reporting breathlessness. The severity, however, was greater in the AL group, with more patients describing New York Heart Association (NYHA) class III/IV symptoms and >50% of ATTRwt patients describing NHYA class I/II symptoms. The variety of presenting symptoms was much wider in the ATTRwt group than among the AL patients. A history of arrhythmia was common; 43.4% had a history of atrial fibrillation (AF), which was the presenting complaint in 10% of patients. AF was more common in the ATTRwt group than among the AL patients. Eight percent of ATTRwt patients were found to have amyloid incidentally on routine testing or at the time of an operation for a different complaint; this feature was not seen in AL amyloidosis. TTR patients diagnosed by cardiac biopsy as opposed to other tissues were younger (median age at diagnosis, 72 versus 77 years; *P*≤0.01), with a lower NT pro‐BNP (median, 295 versus 540 pmol/L; *P*=0.03). Although there was a higher mitral valve E/A ratio (median 2.7 versus 1.6; *P*=0.01) in the cardiac biopsy group all other measures of diastolic dysfunction (IVRT, E/Eʹ, and mitral valve deceleration time) were not significantly different, implying no real difference in overall diastolic function.

**Table 1.** Baseline Patient Characteristics

	AL (n=36)	ATTRwt (n=99)	*P* Value
Male, n (%)	25 (69.4)	88 (88.8)	0.01
Age at diagnosis, years (Q1, Q3)	63.0 (56.6, 65.8)	73.0 (69.5, 78.2)	<0.001
Age at symptom onset, years (Q1, Q3)	60.2 (53.8, 65.2)	70.9 (67.7, 74.1)	<0.001
Age at death (Q1, Q3)	63 (56, 67)	77 (74, 81)	<0.001
NT pro‐BNP, pmol/L (Q1, Q3)	714.0 (427.5, 1573.0)	317.5 (212.3, 909.3)	<0.001
NT pro‐BNP (age ≤70), pmol/L (Q1, Q3)	633 (412, 1073)	293 (227, 404)	
NT pro‐BNP (age >70), pmol/L (Q1, Q3)	2127 (1498, 2755)	440 (244, 794)	
Troponin T, ng/mL (Q1, Q3)	0.05 (0.02, 0.1)	0.04 (0.02, 0.05)	0.3
Positive troponin T, n (%)	19 (52.7)	51 (51.5)	
Hb, g/dL (Q1, Q3)	13 (12.4, 13.6)	14.8 (12.8, 14.8)	0.005
Albumin, g/L (Q1, Q3)	41 (39, 43)	44 (41.3, 46.0)	<0.001
Bilirubin, μmol/L (Q1, Q3)	14 (11, 24)	15 (13, 28)	0.2
ALP, U/L (Q1, Q3)	95 (72, 148)	106 (77, 138)	0.5
GGT, U/L (Q1, Q3)	75.5 (41.0, 142.3)	111.0 (71.0, 162.3)	0.1
eGFR, mL/min (Q1, Q3)	64 (48, 87)	63 (41, 69)	0.2
24‐Hour urine protein, g (Q1, Q3)	0.39 (0.1, 0.8)	0.1 (0.1, 0.2)	<0.001
Lambda free light chain, mg/L (Q1, Q3)	255 (116, 468)	16 (13, 21)	<0.001
Missing, n (%)	0 (0)	8 (8)	
Kappa free light chain, mg/L	31 (7, 22)	18 (14, 24)	0.01
Missing, n (%)	0 (0)	8 (8)	
Detectable paraprotein (%)	24 (66)	14/93 (15)	<0.001
Missing, n (%)	0 (0)	6 (6)	
Any detectable plasma cell dyscrasia,* n (%)	36 (100)	22/91 (24%)	<0.001
Missing, n (%)	0 (0)	8 (8)	
Supine systolic blood pressure, mm Hg (Q1, Q3)	107 (95, 118)	116 (107, 134)	0.006
Supine diastolic blood pressure, mm Hg (Q1, Q3)	72 (60, 76)	74 (66, 81)	0.1
Orthostatic hypotension, n (%)	7 (19)	9 (9)	0.1
Primary presenting symptom, n (%)			
Breathlessness	29 (80.5)	53 (53.5)	
Atrial fibrillation/flutter	0 (0)	10 (10)	
Edema	0 (0)	8 (8)	
Incidental finding	0 (0)	8 (8)	
Syncope	1 (3)	6 (6)	
Palpitations	0 (0)	3 (3)	
Chest pain	2 (5.5)	3 (3)	
Orthostatic hypotension	0 (0)	2 (2)	
Frank hematuria	0 (0)	2 (2)	
Carpal tunnel syndrome	0 (0)	1 (1)	
Cough	0 (0)	1 (1)	
Diarrhea	0 (0)	1 (1)	
Dizziness	0 (0)	1 (1)	
Chest sepsis	1 (3)	0 (0)	
Lethargy	3 (8)	0 (0)	
NYHA class, n (%)			
I	0 (0)	35 (35)	<0.001
II	14 (38)	26 (26)	
III	17 (47)	25 (25)	
IV	5 (13)	6 (6)	
Missing	0 (0)	7 (7)	
Weight loss, n (%)	14 (38)	18 (18)	0.02
History of atrial fibrillation, n (%)	6 (16)	43 (43)	0.004
History of ischemic heart disease, n (%)	4 (11)	27 (27)	0.06
Pacemaker, n (%)	2 (5.5)	13 (13)	0.4
Previous normal coronary angiogram, n (%)	8 (22)	12 (12)	0.2
Previous coronary artery bypass graft, n (%)	0 (0)	8 (8)	0.1
History of chest pain, n (%)	3 (8)	14 (14)	0.6
History of carpal tunnel syndrome, n (%)	3 (8)	48 (48)	<0.01
History of lower‐limb neuropathy, n (%)	3 (8)	9 (9)	1.00
Macroglossia or bruising, n (%)	9 (25)	0 (0)	<0.001

AL indicates light‐chain amyloidosis; ATTRwt, wild‐type transthyretin amyloidosis; NT pro‐BNP, N‐terminal pro‐B‐type natriuretic peptide; Hb, hemoglobin; ALP, alkaline phosphatase; GGT, Gamma‐glutamyl transpeptidase; eGFR, estimated glomerular filtration rate; NYHA, New York Heart Association.

* Either a detectable paraprotein or an abnormal free light‐chain ratio.

A history of carpal tunnel syndrome was significantly more common in the ATTRwt group than in the AL group (48.5% versus 8.3%); in the AL group 2 patients had symptoms of carpal tunnel at the time of diagnosis, and 1 had a release a year before diagnosis. In the ATTRwt group 12 patients had symptoms of carpal tunnel syndrome at the time of diagnosis, and 40 had previous release operations a median of 8 years (Q1, Q3: 3, 10 years) before diagnosis.

### Baseline Biochemical Evaluation

On univariate analysis (Table 1), there were no major differences in biochemical markers of liver function, consistent with the SAP scans, which showed no cases of hepatic amyloid deposits in either group. There was significantly more proteinuria in the AL group, perhaps from renal amyloid involvement, which was below the threshold of detection by SAP scintigraphy. There was no difference in eGFR in patients with proteinuria compared with those without. Three patients with ATTRwt had underlying renal diseases associated with >1 g of proteinuria; all other patients had <1 g of proteinuria at baseline. The most significant difference on univariate analysis was in NT pro‐BNP measurements. There was a median of 714 pmol/L in the AL group and 317.5 pmol/L in the ATTRwt group (*P*<0.001). Evidence of a plasma cell dyscrasia was found in all patients with cardiac AL but also as an incidental finding in 24.1% of patients with ATTRwt (*P*<0.001).

### Baseline Cardiac Investigations

On univariate analysis of ECG features, significantly more patients were in AF or atrial flutter in the ATTRwt group, and more patients had AV conduction abnormalities (Table 2). Low QRS complexes were only seen in 27.3% of the AL patients and 12.9% of the ATTRwt patients. There were significant differences between the 2 groups in several echocardiographic features (Table 3). On univariate analysis, patients with ATTRwt had significantly thicker‐walled hearts than those in the AL group. Both groups had predominantly diastolic dysfunction, most commonly grade III/IV, indicating a restrictive filling pattern. The E/Eʹ was higher in the AL group (median [Q1, Q3], 21.76 [15.7, 26.06] versus 15.81 [12.37, 17.91]; *P*≤0.001). Mitral valve deceleration time was longer in the ATTRwt group (mean, 191.2±59.35 versus 147.9±42.46; *P*≤0.001; CI, 20.42 to 66.26), and the IVRT was also higher in the ATTRwt group (mean, 87.21±25.41 versus 74.35±20.74; *P*=0.01; CI 2.68 to 23.03). Both groups had relatively preserved ejection fractions.

**Table 2.** Electrocardiography at Baseline

	AL (n=34)	ATTRwt (n=93)	*P* Value
Rhythm, n (%)			
Sinus	28 (82)	42 (45)	0.002
Atrial fibrillation	4 (11)	36 (38)	
Atrial flutter	0 (0)	7 (7.5)	
Paced	2 (5)	8 (8)	
Mean voltage leads II/III/AVF (mm)	5.4±3.6	2.9±2.9	0.4
Mean voltage leads V4/V5/V6 (mm)	11.5±5.5	13.5±4.7	0.2
Low QRS complexes* (N/%)	9 (27)	11 (13)	0.2
Any AV conduction abnormality,* n (%)	14 (43)	50 (58)	0.2
Right bundle branch block	1 (3)	14 (16)	
Left bundle branch block	2 (6)	17 (20)	
First‐degree heart block	5 (15)	10 (11)	
Bifascicular block	5 (15)	9 (10.5)	
Junctional rhythm	1 (3)	0 (0)	
QT interval, ms	401±71.5	430.1±55.2	0.003
QTcB, ms	596.6±745.0	478.7±53.3	0.2
T‐wave inversion, n (%)	18 (53)	37 (39)	0.2

AL indicates light‐chain amyloidosis; ATTRwt, wild‐type transthyretin amyloidosis; AV, atrio‐ventricular; QTcB, corrected QT.

* Percentage of patients who were not paced are displayed.

**Table 3.** Baseline Echocardiographic Parameters

	AL	ATTRwt	*P* Value
IVSd, cm	1.5±0.2 (n=34)	1.7±0.3 (n=95)	<0.001
LVPWd, cm	1.5±0.2 (n=34)	1.7±0.2 (n=95)	<0.001
LVIDd, cm	4.2±0.4 (n=34)	4.4±0.6 (n=95)	0.1
LV ejection fraction, %	47.8±12.6 (n=34)	46.6±12.8 (n=95)	0.9
E/A ratio	2.6±1.3 (n=26)	2.4±1.0 (n=66)	0.9
E/E^I^ (IQR)	21.8 (15.7, 26.1) (n=31)	15.8 (12.4, 17.9) (n=86)	<0.001
MVdecT, ms	147.9±42.5 (n=31)	191.2±59.4 (n=91)	<0.001
IVRT, ms	74.4±20.7 (n=31)	87.2±25.4 (n=79)	0.01
TDI wave lateral, ms	0.06±0.02 (n=20)	0.06±0.05 (n=70)	0.4
Grade of diastolic dysfunction, n (%)*	(n=29)	(n=76)	
Normal	0		0.4
I	2 (7)	10 (13)	
II	5 (17)	19 (25)	
III/IV	22 (76)	47 (62)	

AL indicates light‐chain amyloidosis; ATTRwt, wild‐type transthyretin amyloidosis; IVSd, interventricular septal thickness in diastole; LVPWd, left ventricular posterior wall thickness in diastole; LV, left ventricular; E/A, doppler of transmitral inflow velocities measured at the tip of the mitral leaflets; E/E^I^, left sided ventricular filling pressures; IQR, interquartile range; MVdecT, mitral valve deceleration time; IVRT, isovolumetric relaxation time; TDI, tissue Doppler imaging.

* Because of varying times at which echocardiography was performed, not all measures are reported in all patients.

### Distinguishing Between Patients With Isolated AL Amyloidosis and ATTRwt

**Figure 1.**
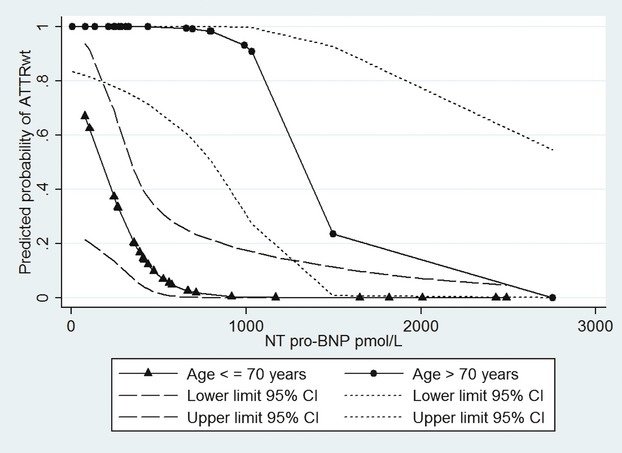
Predicted probability of wild‐type transthyretin amyloidosis (ATTRwt) in patients ≤70 or >70 years with a detectable plasma cell dyscrasia by N‐terminal pro‐B‐type natriuretic peptide (NT pro‐BNP).

We sought to identify baseline characteristics that could distinguish between the 2 diagnoses. All patients in the AL amyloidosis group had a detectable underlying plasma cell dyscrasia, indicating that if no plasma cell dyscrasia could be identified in this cohort, the diagnosis was ATTRwt. No patients with ATTRwt had evidence of macroglossia, which was purely an indicator of AL amyloidosis. A logistic regression model was used on the remaining 47 patients (25 patients with AL amyloidosis and 22 patients with ATTRwt) who had a detectable plasma cell dyscrasia and no macroglossia. There were no clinically relevant differences in baseline characteristics in the excluded patients when stratified by amyloid type. NT pro‐BNP and age at diagnosis were both associated with risk of ATTRwt. NT pro‐BNP was higher in patients with AL amyloidosis. The odds of having ATTRwt reduced by 0.1% for every pmol/L unit increase in NT pro‐BNP (*P*=0.04). Patients with ATTRwt were older, with the odds of ATTRwt increasing by 102% for every 1‐year increase in age (*P*=0.02). A further logistic regression analysis that replaced actual age at diagnosis with age at diagnosis as a binary variable (27 patients aged ≤70 years and 20 patients aged >70 years) indicated that both the binary age variable and NT pro‐BNP at baseline were significantly associated with diagnosis (*P*=0.009 and *P*=0.03, respectively). Figure 1 shows the probability of having ATTRwt amyloidosis in patients who have isolated cardiac amyloidosis without macroglossia or easy bruising with a detectable plasma cell dyscrasia in patients above or below 70 years of age according to the NT pro‐BNP at baseline. Patients ≤70 years old were more likely (*P*>0.5) to have ATTRwt than AL if the NT pro‐BNP was <183 pmol/L, and patients >70 years old with an NT pro‐BNP of <1420 pmol/L were more likely to have ATTRwt. Using this combination of age and NT pro‐BNP, the positive predictive value was 90.0% and negative predictive value was 85.2%, with a positive likelihood ratio of 10.2 and negative likelihood ratio of 0.19. The area under the ROC curve was 0.98 (CI, 0.95 to 1.0), sensitivity 83.3%, and specificity 92%.

### Patient Survival

**Figure 2.**
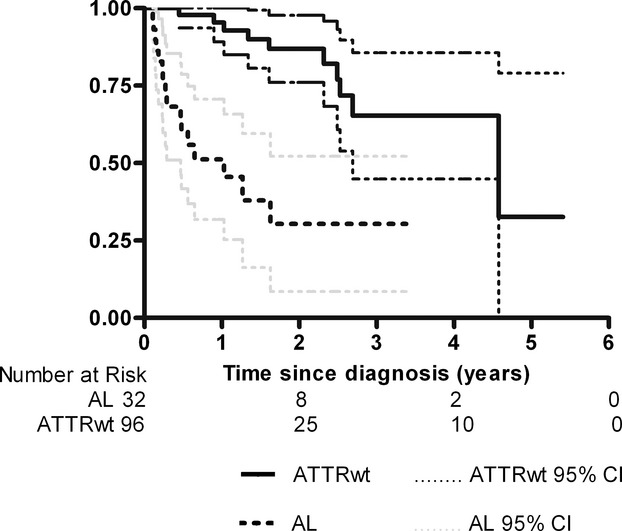
Patient survival from diagnostic biopsy. Median survival of patients with wild‐type transthyretin amyloidosis from diagnostic biopsy is 2.71 years compared with 0.87 years for patients with isolated cardiac AL amyloidosis. Overall survival is significantly longer in the ATTRwt group (*P*=0.002 log‐rank [Mantel–Cox] test). AL indicates light‐chain amyloidosis; ATTRwt, wild‐type transthyretin amyloidosis.

**Figure 3.**
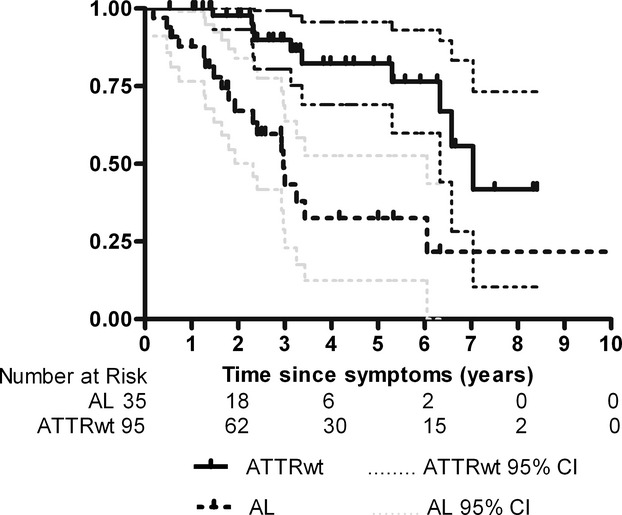
Patient survival from onset of symptoms. Median survival of patients with wild‐type transthyretin amyloidosis from onset of symptoms is 6.07 years compared with 1.7 years for patients with isolated cardiac AL amyloidosis. Overall survival from symptoms is significantly longer in the ATTRwt group (*P*≤0.0001 log‐rank [Mantel–Cox] test). AL indicates light‐chain amyloidosis; ATTRwt, wild‐type transthyretin amyloidosis.

Survival from diagnosis in patients with ATTRwt was significantly longer than for the cardiac‐isolated AL group (log‐rank test, *P*=0.001). Median survival for patients with ATTRwt was 2.71 years and for cardiac AL amyloidosis patients, survival was 0.87 years (Figure 2). Median survival from the onset of symptoms was much longer than from diagnosis in the ATTRwt group: 6.07 years compared with 1.7 years in the cardiac AL group (log‐rank test, *P*≤0.0001; Figure 3).

Factors at baseline that were associated with poorer survival in the ATTRwt patients on univariate analysis were: a positive troponin (median survival, 1.8 versus 4.6 years; log‐rank test, *P*=0.01) a pacemaker (median survival, 1.3 versus 4.06 years; log‐rank test, *P*=0.002), and NYHA class (median survival: class I, 4.58 years; class II, 4.06 years; class III, 2.08 years; and class IV, 1.31 years; log‐rank test for trend, *P*<0.001). These factors remained significantly associated with death in a Cox proportional hazards multivariable analysis; however, because of the sample size and limited number of events, the confidence intervals showed inadequate precision, and therefore no absolute conclusions could be drawn from the current patient numbers.

Thirty‐two patients in the ATTRwt group died. The primary causes of death reported on the death certificates were as follows: 17 from congestive cardiac failure and 1 each from complete heart block, hypertensive heart disease, ischemic heart disease, ischemic stroke, old age, multiorgan failure, renal failure, acute myeloid leukemia, mesothelioma, metastatic lung cancer, urinary tract infection, and pneumonia. The cause of death was unknown to us in 3 patients. Only 2 patients in the AL amyloidosis group developed extracardiac amyloid; 1 patient had symptoms of progressive peripheral neuropathy, and 1 patient developed end‐stage renal failure. Twenty‐one (58%) died before their 6‐month follow‐up appointment.

## Discussion

ATTRwt amyloidosis is a slowly progressive disease of the elderly whose incidence is unknown. ATTRwt amyloid has been shown to deposit in the carpal tunnel.^20^ A recent series has reported ATTRwt deposits in 34% of patients with idiopathic carpal tunnel syndrome^21^ Connors et al^22^ recently reported that carpal tunnel syndrome was rare in patients with ATTRwt cardiac amyloid. It has been suggested that systemic deposition of TTR occurs years before the onset of cardiac dysfunction.^23–24^ In our series, 48% of ATTRwt patients gave a history of carpal tunnel syndrome that preceded the onset of clinical symptoms of heart failure in 77%, a median of 8 years before the diagnosis of amyloidosis being made, consistent with a slowly evolving disease. ATTRwt can be found in other tissues aside from the myocardium and wrist.^25–27^ Almost 4% of our patients presented with frank hematuria or an incidental finding on bladder histology. No patients have had amyloid detectable on radiolabeled ^123^I SAP scintigraphy.

The apparent increase in the disease over the last couple of years reflects an increase in the number of referrals to our center. Although the total number of amyloid referrals has risen, the proportion referred from cardiologists has vastly increased. Thirty‐seven percent of cases had amyloid suspected on the basis of CMR findings. The use of sophisticated cardiac imaging techniques such as CMR in elderly patients is now more readily available, and as the population ages, the prevalence of the disease may well be increasing.

Studies describing the natural history of the disease have been limited to small numbers.^11^ This long‐term follow‐up study of 102 patients with ATTRwt amyloidosis is, to our knowledge, the largest study to date. Patients predominantly presented with heart failure, although breathlessness was generally less severe than in cardiac AL amyloidosis despite greater left ventricular (LV) wall thickness. Patients with ATTRwt amyloidosis had more arrhythmias than those with AL and were more likely to have required pacing before diagnosis. Our findings are consistent with those reported by Rapezzi et al^11^ where patients with ATTRwt were found to have the greater LV wall thicknesses than those with cardiac AL and hereditary TTR amyloidosis and a higher incidence of left bundle branch block. Low QRS complexes are widely considered a marker of diagnosis of cardiac amyloidosis. In this study the prevalence of this was lower than previously described.^28^ Although low‐voltage QRS complexes on the ECG remain a potential indicator of cardiac amyloid, it is important to note that normal‐size complexes should not deter physicians from considering a diagnosis of amyloid.

A marked survival difference between patients with ATTRwt and cardiac AL amyloidosis has been reported before.^11,22^ Median survival in patients with ATTRwt was 2.71 years from diagnosis compared with 0.87 years in the AL group. This is even more striking when survival from symptom onset is considered; the median survival in the ATTRwt group was 6.07 years, and this may be a more useful indicator for patients who have been picked up early on in the course of the disease or whose cardiac amyloid is an incidental finding.

The presence of a pacemaker was associated with shorter survival from diagnosis. Clearly this does not necessarily imply causality; indeed, it seems likely that patients with conduction abnormalities necessitating a pacemaker are at higher risk of sudden cardiac death. A recent study in familial amyloid polyneuropathy reported that prophylactic pacemakers prevented major cardiac events in 25% of patients.^29^ There is no specific literature on the role of implantable cardiac defibrillators in ATTRwt amyloidosis, but in the AL type it seems that very careful patient selection is required, as most sudden cardiac death is from pulseless electrical activity that is not amenable to cardioversion.^30^

A positive troponin T at baseline was associated with poorer survival. Suhr et al^31^ reported NT pro‐BNP to be a sensitive marker for diagnosing ATTR cardiomyopathy in hereditary Val30Met patients. Recent data from Russo et al suggested that both troponin and BNP are prognostic indicators in ATTR amyloidosis. Troponin appeared to be a stronger predictor (HR, 2.2; 95% CI, 1.4 to 3.5; *P*≤0.01) compared with BNP (HR, 1.2; 95% CI, 1.1 to 1.4; *P*<0.01).^32^ Interestingly, higher baseline NT pro‐BNP in our cohort was not associated with poorer survival. Survival worsened with increasing NYHA class on univariate analysis. In our study echocardiographic features such as wall thickness and degree of diastolic or systolic dysfunction did not affect survival. Although LV wall thickness has been reported to be associated with survival in patients with cardiac amyloidosis,^11^ we were unable to draw any definite conclusions in our patients with ATTRwt because of inadequate numbers for analysis.

The combination of transthyretin amyloid on endomyocardial biopsy and wild‐type TTR gene sequencing is the gold standard for diagnosing ATTRwt. Although endomyocardial biopsy carries a low reported risk of complications of between 1% and 2%,^33^ some elderly patients are not keen to undergo such an invasive investigation. Tissue from screening biopsies such as abdominal fat and the rectum can be helpful in diagnosing amyloid; however, false‐negatives can occur because of patchy amyloid deposition, and Connors et al^22^ recently reported that only 27% of fat aspirates in ATTRwt were positive. When histological confirmation is not possible, clinicians must rely on a combination of clinical history, imaging, and biochemical markers to come to a diagnosis. 99mTc‐DPD scintigraphy has been shown to be a sensitive imaging technique in diagnosing cardiac transthyretin amyloidosis.^34–36^ Studies are currently limited by small numbers, but the technique appears to be highly specific for transthyretin amyloidosis,^37^ and it is emerging as a helpful diagnostic tool; however, the availability of the technique is currently limited to centers within Europe.

The prevalence of MGUS in the general population is 5.3% among those >70 years, rising to 7.5% among those ≥85 years.^38^ Twenty‐four percent of patients with proven ATTRwt in this series were found to have a detectable plasma cell dyscrasia. This high prevalence highlights both that highly sensitive techniques can detect incidental low‐grade clones in a large proportion of the elderly, although obviously this may also induce bias with amyloidosis sought most diligently in patients with a detectable clonal excess, and that the presence of a clone cannot be used to diagnose AL‐type amyloidosis without further confirmation. Clearly, the converse is also a concern, although in this series all the patients with isolated cardiac AL amyloidosis did have a detectable plasma cell dyscrasia. Indeed, in our center cardiac biopsy‐proven isolated cardiac amyloidosis accounts for only 1.84% of patients with systemic AL amyloidosis; although it has been reported that 6% of patients with AL amyloidosis have no detectable clone,^1^ our recent experience from the prospective ALCHEMY cohort^39^ suggests that only 1 of 494 new patients with AL amyloidosis seen at our center had no evidence of a plasma cell clone on serum and urine investigations. One would therefore predict that it is extremely rare for a patient with AL amyloidosis to present with isolated cardiac disease and no detectable clone, although this is theoretically possible.

A much more frequent clinical problem is differentiating between the ATTRwt and AL types in elderly patients who have isolated cardiac amyloid and an abnormal clone. Cardiac biopsy remains the only definitive method of distinction, and some patients are not prepared to undergo this invasive procedure. In this study, despite excluding patients with isolated cardiac amyloid and symptoms that are pathognomonic for AL amyloidosis such as macroglossia or easy bruising,^1^ it was possible to estimate the risk of ATTRwt amyloid in patients with a detectable monoclonal protein by using a combination of age and NT pro‐BNP. NT pro‐BNP is dependent on factors such as renal function, arrhythmias, and fluid balance. Although these factors all display colinearity with NT pro‐BNP, they were discarded on logistic regression analysis, and NT pro‐BNP remained a strong predictor of the diagnosis of either an AL or ATTRwt assessment of a patient with amyloidosis.

## Conclusions

**Figure 4.**
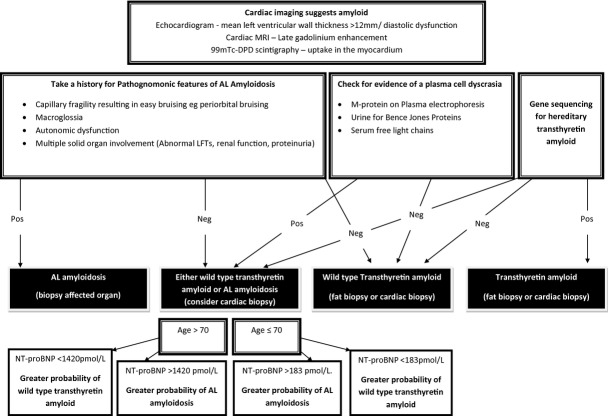
Diagnostic algorithm for patients presenting with suspected cardiac amyloidosis based on cardiac imaging. Initial investigations should include a comprehensive patient history and examination, investigations for an abnormal clone, and genotyping for hereditary transthyretin amyloidosis. MRI indicates magnetic resonance imaging; AL, light‐chain amyloidosis; NT pro‐BNP, N‐terminal pro‐B‐type natriuretic peptide.

An accurate diagnosis of amyloid type in patients with apparently cardiac‐isolated disease is paramount. Cardiac AL amyloidosis and ATTRwt are separate diseases with very different outcomes and treatment. Survival in ATTRwt is far superior to cardiac AL amyloidosis, and chemotherapy is inappropriate in isolated ATTRwt. Troponin T, the need for a pacemaker, and severe functional impairment (NYHA class IV symptoms) are associated with poor outcome. We have generated an algorithm that outlines the diagnostic process once a patient is diagnosed with cardiac amyloid and uses a patient's age at diagnosis and level of NT pro‐BNP to help to distinguish between the 2 disease groups in patients with isolated cardiac disease and a detectable plasma cell dyscrasia (Figure 4); however, the gold standard is a histological diagnosis.
